# Positive versus negative mental health in emerging adulthood: a national cross-sectional survey

**DOI:** 10.1186/1471-2458-14-1238

**Published:** 2014-12-01

**Authors:** Regina Winzer, Frank Lindblad, Kimmo Sorjonen, Lene Lindberg

**Affiliations:** Department of Public Health, Karolinska Institutet, Stockholm, Sweden; Department of Neuroscience, Uppsala University, Uppsala, Sweden; Stress Research Institute, Stockholm University, Stockholm, Sweden; Department of Clinical Neuroscience, Karolinska Institutet, Stockholm, Sweden

**Keywords:** Positive and negative mental health, General health questionnaire, National health survey, Adolescence, Young adulthood

## Abstract

**Background:**

The dual continuum model suggests that positive mental health not only implies the absence of mental illness, but also constitutes an entity of its own. Measures that encompass both positive and negative mental health in young adults are rare. Thus, we assessed whether dimensions corresponding to positive and negative mental health could be identified in a sample of young individuals. Additionally, we explored how such dimensions were associated with potential health-related factors.

**Methods:**

We obtained data from the Swedish National Public Health Survey 2004–2009 (23,394 women, 18,274 men, aged 16–29 years). We used exploratory factor analysis (EFA) to identify relevant factors revealed by the 12-item General Health Questionnaire (GHQ-12) and confirmatory factor analysis (CFA) to verify the factor structure. We tested the significance of the difference between effects of potential health-related factors on positive mental health (PMH) and negative mental health (NMH).

**Results:**

The EFA for the GHQ-12 revealed a two factor model with negative items that had high positive loadings on one factor and lower negative loadings on the other factor. The positive items had loading trends that were opposite those of the negative items. The fit of this model was supported by the CFA, which yielded a significantly better match than a unidimensional model. When we investigated the associations between GHQ-scores and potential predictors of health, we found that most potential predictors had significant and opposing effects on both PMH and NMH; with the strongest effects from suicidal ideation and perceived humiliation.

**Conclusions:**

Our results could be seen to indicate that positive and negative mental health are distinct and complementary constructs. Still, the results of our factor analysis may specifically reflect the wording of the items. We conclude that the GHQ-12 is an appropriate tool for its original purpose, to detect “psychiatric morbidity”. More refined measures, including predictors of health, are needed to assess PMH and validate the bidimensionality hypothesis.

## Background

Positive and negative mental health are often vaguely defined. Moreover, these constructs are frequently conflated, as mental health research, mental health initiatives, and mental health surveys often focus purely on negative mental health, e.g. symptoms of anxiety, depression and psychological distress. One common way to conceptualize these theoretical constructs is to view them as two poles of a continuum [1]. However, proponents of positive psychology have emphasized the discontinuity between positive mental health and mental ill-health, suggesting that (positive) mental health both implies the absence of mental ill-health and constitutes a distinct entity [2, 3]. Some of the processes that effect positive mental health are quite distinct from those concerning mental ill-health [4]. Keyes termed this approach “the dual continuum model”, stating “mental health and mental illness belong to two separated but correlated dimensions among the population” [5]. Keyes also introduced the terms “flourishing” and “languishing”, which reflect high and low levels, respectively, of well-being and functioning. These states can exist in both the presence and absence of mental illness [6]. Similarly, Keyes suggested “curing or eradicating mental illness will not guarantee a mentally healthy population” [7]. Furthermore, Keyes reported that languishing adults report the same degree of health-related limitations in daily living and levels of psychosocial functioning compared with mentally ill adults with moderate or flourishing mental health [8]. Thus, a national health strategy should continue to focus on treating and preventing mental illness while simultaneously promoting a state of flourishing in people free from mental illness but in lack of positive mental health [7].

Several different instruments/scales have been used to explore and possibly confirm the rationale of the dual continuum model [9–12]. One popular instrument that has been well validated is the General Health Questionnaire (GHQ) [13]. Several versions are available, with 60, 30, 28, and 12 items. Currently, the 12-item version is most frequently used. The GHQ was originally intended to be used as a one-dimensional scale for assessing “psychiatric morbidity” in clinical and community settings [13]. Health surveys commonly use the GHQ with a score of >2 or >3 indicating negative mental health [14, 15]. Various surveys have examined the positive and negative items of the GHQ-12 and GHQ-30 separately [16–19]. These studies indicate that the two classes of items can be used as separate but correlated scales reflecting “positive and negative mental health” [4]. The positive and negative dimensions of the GHQ-12 were tested in two population studies in the UK. The researchers identified two factors, one corresponding to “symptoms of mental disorder” and the other to “positive mental health”. Additional analyses showed that these factors were associated with age, gender, employment status, housing, and household composition in unique ways [20]. Huppert and Whittington reported that differences between levels of positive mental health and mental ill-health were associated with demographic, health-related, and social factors [4]. Specifically, physical illness, disability, and lack of social support were strongly associated with negative mental health but not with positive mental health.

National health studies frequently focus on predictors of mental ill-health, while they rarely examine predictors of positive mental health [15, 21, 22]. Studies in which predictors of positive mental health and ill-health are analyzed simultaneously have usually focused on children [23], adolescents [24] or the entire population [25, 26]. To the best of our knowledge, no earlier population-based study has concurrently focused on potential predictors of positive and negative mental health in an age-extended youth group.

Sociological and demographical studies have shown that the length of the transition from adolescence to young adulthood has increased considerably during the last decades [27, 28]. Simultaneously, in some societies, this group seems to have developed a greater incidence of mental ill-health compared with other age groups [29–31], indicating that further studies are necessary. Current research indicates that in young people, socioeconomic, educational, and psychological statuses, as well as social context and health behavior, influence various adverse mental health outcomes and health inequities in later life [32–35]. Thus, a comparative analysis of potential predictors of positive mental health versus negative mental health in young people may be of particular interest. Hence, we focused on individuals aged 16 –29 years.

Our first aim was to determine whether we could identify dimensions corresponding with positive and negative mental health in a sample of young individuals. Our second aim was to study how each dimension was related to demographic, social, and health factors, and to explore any differences and similarities between these associations.

## Methods

### Participants and procedures

We used data from the Swedish National Public Health Survey for the years 2004 to 2009. The survey is conducted annually via post and the internet, and is carried out by Statistics Sweden in collaboration with healthcare regions and districts. The whole procedure is coordinated by the Swedish National Institute of Public Health. Thus, the study population comprised six annually selected national samples of 10,000 (2005, 2006, 2007) or 20,000 (2004, 2008, 2009) persons aged 16–84 years (18–84 years in 2004). Participating county councils, regions, and municipalities varied by year. The samples were randomly selected, or selected using various stratified sample criteria (e.g. municipality, age). In total, 134,563 women and 113,724 men responded. Of these, 23,394 women and 18,274 men were 16–29 years of age.

We used all available data for questions concerning health, social factors, and lifestyle (for more details see [36]). The data were in the form of self-administered questionnaires completed between 2004 and 2009, and linked to the registry data from Statistics Sweden. Information about native country and citizenship was retrieved from the registry data. The respondents were informed about the data linkage and confidentiality was ensured. Three reminders were sent out if the questionnaires were not returned in time. The response rate varied from 57% to 61%, depending on the year. The Department of Data Inspection and the Research Ethical Committee at the National Board of Health and Welfare (20031208) approved the consent procedure for The Swedish National Health Survey for all participants, irrespective of age. The Regional Ethical Review Board of the Stockholm Committee (No 2007/1021-31/3) approved the present study. As of January 1, 2004, Swedish legislation (SFS 2003:460, §16, §17, §18) deemed that parental consent would no longer be required for minors between 15 and 18 years of age to take part in surveys, when they are made aware of the research implications.

### Study variables

#### Positive mental health and negative mental health

We used the General Health Questionnaire, GHQ-12 [13], to assess positive and negative mental health. The items about everyday functioning referred to the past several weeks.

The items included in the GHQ-12 are listed in Table 1. Items 1, 2, 4, 5, 7, and 12 had four options: ‘More/better than usual’, ‘Same as usual’, ‘Less/worse than usual’ and ‘Much less/much worse than usual’. Items 3, 6, 8, 9, 10, and 11 had the following four options: ‘Not at all’, ‘Not more than usual’, ‘More than usual’ and ‘Much more than usual’. The responses were coded as ordinal variables.

**Table 1:** **Exploratory factor analysis factor loadings for the GHQ-12 items with one and two factors, including orthogonal (varimax) and oblique (geomin) rotation**

		Orthogonal		Oblique	
Item	One factor	Factor 1	Factor 2	Factor 1	Factor 2
GHQ 1: Have you recently been able to concentrate on daily tasks? (pos)	−.676	.562	−.434	.556	−.211
GHQ 2: Have you recently been able to enjoy your normal day-to-day activities? (pos)	−.653	.692	−.311	.764	.007
GHQ 3: Have you recently lost much sleep due to worrying? (neg)	.625	−.170	.646	.016	.678
GHQ 4: Have you recently been able to face up to your problems? (pos)	−.614	.647	−.309	.707	−.015
GHQ 5: Have you recently felt that you were playing a useful part in things? (pos)	−.544	.647	−.205	.744	.109
GHQ 6: Have you recently been feeling unhappy or depressed? (neg)	.823	−.334	.768	−.148	.733
GHQ 7: Have you recently felt capable of making decisions about things? (pos)	−.595	.625	−.291	.686	−.006
GHQ 8: Have you recently been losing confidence in yourself? (neg)	.868	−.264	.848	−.032	.867
GHQ 9: Have you recently felt yourself to be constantly under strain? (neg)	.696	−.159	.736	.061	.791
GHQ 10: Have you recently been thinking of yourself as a worthless person? (neg)	.862	−.236	.859	.008	.896
GHQ 11: Have you recently felt that you couldn’t overcome your difficulties? (neg)	.797	−.285	.768	−.086	.761
GHQ 12: Have you recently been feeling reasonably happy, all things considered? (pos)	−.657	.545	−.430	.535	−.215
χ^2^	43183	9255		9255	
Df	54	43		43	
CFI	.913	.981		.981	
TLI	.894	.972		.972	
RMSEA	.139	.072		.072	

CFI, comparative fit index; Df, TLI, Tucker–Lewis index; RMSEA, root-mean-square error of approximation.

Model fit at the bottom.

To analyze possible associations and the strengths of potential predictors, we examined the survey for variables related to socio-demography, support, lifestyle, violations, and suicidal ideation and behavior. See Table 2 for a detailed description of the included variables. The variables are summarized below.

**Table 2:** **Potential predictors and associated response options**

**Sociodemographic factors**	
Country of birth (register data)	Swedish, Nordic, European, Non-European
Housing (How do you live?)	Own, Rented, Student’s room/lodging
Occupational status (Your current occupation?)	Student, Employed, Unemployed, Sickness benefit/disability pension, Other activities
Economic strain (Have you had difficulties paying for food, rent, bills etc. during the last 12 months?)	No, Yes (yes once/yes several times)
**Support, Trust, Participation**	
Emotional support (Do you have anybody to share your inner feelings with and confide in?)	Yes, No
Practical support (Can you get help from another person/other persons if you have a practical problem or are ill?)	Yes (yes always/yes most of the time), No
Interpersonal trust (Do you believe that you can generally trust most people?)	Yes, No
Community trust (What is the level of trust that you place in the following institutions/politicians in your society?)	Summary index = Very high, High, Low, Very low
Participation (Have you taken part in the following activities during the past 12 months?)	Summary index = High, Moderate, Low, None
**Healthy eating, Physical activity**	
Healthy eating (Consumption of vegetables and fruit)	Summary index = Low consumption, High consumption
Physical activity (How much have you been physically active (including leisure activities) during the last 12 months?)	Sedentary leisure, Moderate exercise during leisure, Moderate regularly exercise during leisure, Regularly exercise and training
**Alcohol, Gambling, Smoking**	
Alcohol consumption (Have you been drinking alcohol during the last 12 months?)	Summary Index = Yes (≥4 times/week, 2–3 times/week, 2–4 times/month, once/month or more seldom), No (never)
Risky alcohol consumption (3 questions)	Summary index = Low consumption, High consumption
Gambling (Have you been bought lottery tickets or bet money on games during the last 12 months?)	No, Yes
Risky gambling (3 questions)	Summary index = No risk, High risk
Smoking (Do you smoke every day?)	No, Yes
**Violations**	
Humiliation (Have you been treated in a way that led to feelings of humiliation during the past three months?)	None (no), Some (Yes, sometime), Frequent (Yes, several times)
Threat (Have you been exposed to threats or threats of violence that made you scared during the last 12 months?)	Yes, No
Violence (Have you been subject to physical violence during the past 12 months?)	Yes, No
**Suicidal expressions**	
Suicide ideation (Have you ever considered attempting suicide?)	No, Yes once, Yes several times
Suicide attempt (Have you ever attempted suicide?)	No, Yes once, Yes several times

We created an index for *community trust* using responses to questions about how much the respondent trusted the health care system, schools, police, social services, employment services, Swedish Social Insurance Administration, courts, parliament, and politicians [37]. The summarized scores for the responses were categorized as being “Very high” (very high or rather high for all 10 questions), “High” (very high or rather high for 8 or 9 questions), “Low” (low or no trust for 3–5 questions) and “Very low” (low or no trust for 6–10 questions). The reliability of *community trust* has previously been reported to be high [37], and the Cronbach α = 0.84 in this study.

We created the *participation* index using responses to questions about respondent participation (in the last 12 months) in any of the following: a study circle/course at work, a study circle/course during leisure time, union meetings, other meetings with associations, theatre/cinema, art exhibitions, church, sporting events, writing a letter to a newspaper, taking part in political manifestations, public events such as night clubs or dancing, meeting with relatives, and private parties. The summarized scores for the responses were categorized as being “High” (7–13 activities), “Moderate” (2–6 activities), “Low” (1 activity), and “None” (none of the listed activities).

There were two questions concerning *healthy eating*: “How often do you eat vegetables and root vegetables?” and “How often do you eat fruits and berries?” The responses for each question were weighted such that 3 times a day or more was coded as 3, twice a day was coded as 2, once a day was coded as 1, 5–6 times a week was coded as 0.8, 3–4 times a week was coded as 0.5, 1–2 times a week was coded as 0.2 and sometimes per month or never was coded as 0.07. The weighted answers from the two questions were then combined to produce a value between 0.07 and 6. The cut-off for dichotomization was set at 5, with a value of <5 categorized as “Low consumption” and ≥5 as “High consumption” [36].

We used three questions from the Swedish version of the Alcohol Use Disorders Identification Test [38] as a measure of *risky alcohol consumption*. The first was “How frequently have you drank alcohol in the past 12 months?” with possible responses of never (0), once per month or less (1), 2–4 times a month (2), 2–3 times a week (3), and 4 times a week or more (4). The second question was “How many glasses of alcohol do you have on a typical day when you are drinking?” One glass corresponded to 330 ml of beer, 100–150 ml of wine, or 40 ml of liquor, with the following possible responses: 1–2 glasses (0), 3–4 glasses (1), 5–6 glasses (2), 7–9 glasses (3) and 10 or more glasses (4). The last question was “How often do you have six or more glasses of alcohol on one occasion?” with the following response options: never (0), less than once per month (1), monthly (2), weekly (3) and daily or almost daily (4). The points were calculated and respondents were categorized as having either low (<8 points for men, <6 for women) or high (≥8 points for men, ≥6 for women) consumption [37, 39].

*Risky gambling* was assessed via the following three items: “How many times during the last 12 months have you a) tried to reduce the frequency of your gambling, b) felt restless and irritated if you have been unable to gamble, and c) lied about how much you have gambled”. The responses were chosen from the following: never (0), 1–2 times (1), and 3 times or more (2). The scores were calculated and a value of 1 was used as the cut-off for risky gambling.

### Data analyses

Data analyses were conducted with M*plus* 7.11 software. To assess whether the GHQ-12 could be used to measure both positive and negative mental health, we used exploratory factor analysis (EFA). Scree plots, eigenvalues, and differences in model fit were used to determine the number of factors. We performed orthogonal (varimax) and oblique (geomin) rotation analyses. We also conducted confirmatory factor analysis (CFA). We used two indices of incremental fit—the Tucker–Lewis index (TLI) and the comparative fit index (CFI)—to assess the fit between specified models and the data. We considered a value > .95 on these indices to indicate a close fit. We also used the root-mean-square error of approximation (RMSEA) on which a value <0.05 is considered to indicate a close fit [40]. The GHQ-12 items were defined as ordinal and we used a robust weighted least squares estimator. We used the DIFFTEST function in Mplus to calculate the significance of the difference between the effects on positive mental health and negative mental health.

## Results

The EFA revealed that the amount of explained variance increased from 52.5% to 64.6% when reducing the GHQ-12 items to two factors rather than one factor and the model fit improved significantly (*p* < .001). With three factors the improvement was also significant (*p* < .001, 70.3% explained variance). However, this third factor had eigenvalue <1 (.692), and with orthogonal varimax rotation only one item (number 3) had its highest loading on this factor (with oblique geomin rotation, no item had its highest loading on factor 3). Factor loadings are shown in Table 1, which shows how the symptom and problem oriented (“negative”) items had high positive loadings on the second factor and lower negative loadings on the first factor. In contrast, the capacity oriented (“positive”) items had high positive loadings on the first factor and negative loadings on the second factor.

**Figure 1:**
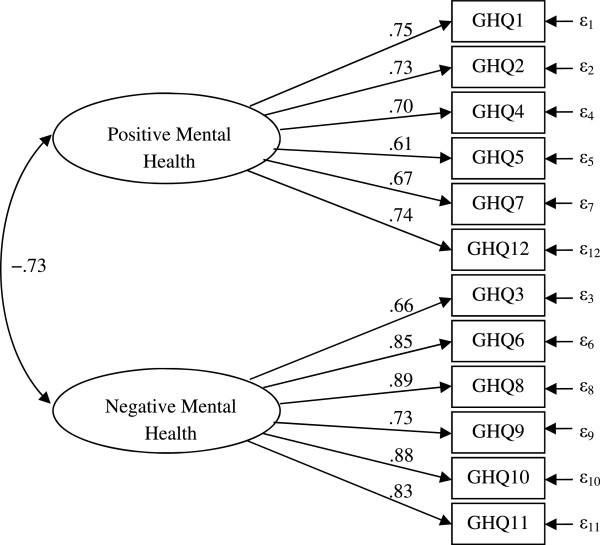
**Parameter values in a confirmatory factor analysis (CFA) where positive mental health and negative mental health are treated as two separate constructs.**

We used a CFA to verify the factor structure. A model where all items were treated as indicators of the same latent variable was a significantly worse fit to the data compared with a model that had two separate latent variables, χ^2^(54) = 43,183, *p* < .001; TLI = .894; CFI = .913; RMSEA = .139; and χ^2^(53) = 12,492, *p* < .001; TLI = .969; CFI = .975; RMSEA = .075, respectively. The fit of the model with two separate latent variables (Figure 1) could be characterized as quite satisfactory according to the criteria presented by Hu and Bentler [40]. The factors were tentatively called “positive mental health” (PMH) and “negative mental health” (NMH).

Table 3 shows the effects of our chosen presumptive predictors on PMH and NMH, both crude and adjusted for one another. We calculated the significance of the difference between the absolute values of these effects. A significant difference indicates that a predictor had a stronger effect (= the value of the effect deviates more from zero) on one of the outcome variables compared with the other.

**Table 3:** **Effects of some predictors on positive (PMH) and negative (NMH) mental health, both crude and adjusted for one another**

	Crude effect ^a^				Adjusted effect ^a^			
Predictor	PMH	NMH	Diff ^b^	χ ^2c^	PMH	NMH	Diff ^b^	χ ^2c^
Female Sex	−.177*	.438*	−.261	395*	−.124*	.308*	−.184	62.8*
Age	−.132*	.002	.130	59.9*	−.206*	.054	.152	23.0*
Country of birth^d^								
Nordic	−.056	.042	.014	1.13	.045	−.013	.032	1.15
European	.053	.068^†^	−.015	0.02	.052	.026	.026	0.13
Other	−.129*	.249*	−.120	19.5*	−.052	.026	.026	1.59
Housing^e^								
Rented	−.136*	.199*	−.063	11.3*	−.008	.052^†^	−.044	5.32^†^
Other	−.068*	.156*	−.088	26.4*	.030	.041	−.011	0.02
Employment^f^								
Employed	.070*	−.216*	−.146	89.0*	.149*	−.230*	−.081	6.11^†^
Unemployed	−.326*	.308*	.018	9.86^†^	−.105^†^	.014	.091	10.7^†^
Sickness benefit/disability pension	−.934*	.976*	−.042	4.97^†^	−.588*	.405*	.183	26.2*
Other	−.019	.036^†^	−.017	0.60	.113*	−.160*	−.047	0.94
Economic strain	−.351*	.569*	−.218	187*	−.072*	.208*	−.136	38.2*
Emotional support	.500*	−.573*	−.073	1.02	.340*	−.350*	−.010	2.47
Practical support	.693*	−.814*	−.121	0.51	.261*	−.224*	.037	3.82
Interpersonal trust	.329*	−.473*	−.144	67.7*	.171*	−.188*	−.017	1.39
Community trust	.303*	−.444*	−.141	18.3*	.046	−.156*	−.110	7.49^†^
Participation	.237*	−.148*	.089	21.3*	.120^†^	−.028	.092	6.09^†^
Healthy eating	.119*	.013	.106	22.5*	.070^†^	.005	.065	3.72
Physical activity	.395*	−.463*	−.068	1.37	.227*	−.215*	.012	3.76
Alcohol cons.	−.092*	.146*	−.054	6.70^†^	−.086^†^	.119*	−.033	1.28
Risky alc. cons.	−.152*	.275*	−.123	49.5*	−.053^†^	.069*	−.016	0.08
Gambling	.031^†^	.051*	−.020	0.47	.168*	.027	.141	4.51^†^
Risky gambling	−.236*	.379*	−.143	9.74^†^	−.090^†^	.216*	−.126	7.79^†^
Smoking	−.165*	.244*	−.079	18.2*	−.026	.011	.015	0.53
Humiliation	−.737*	1.262*	−.525	541*	−.397*	.692*	−.295	56.9*
Threat	−.392*	.618*	−.226	92.0*	−.090^†^	.108*	−.018	1.01
Violence	−.281*	.419*	−.138	26.5*	−.015	.015	.000	1.01
Suicide ideation	−.781*	1.269*	−.488	461*	−.349*	.720*	−.371	107*
Suicide attempt	−.937*	1.418*	−.481	163*	−.075	−.058	.017	1.08

^†^ *p* < .05; *p < .001; ^a^The effects stand for the predicted difference, in *SD,* in mental health between the highest and lowest values of the predictors; ^b^Difference between the absolute values of the effects; ^c^Chi square value for the difference between the effects, a significant value indicates that one of the effects is stronger (deviates more from zero) than the other effect; ^d^Reference category = Sweden; ^e^Reference category = own; ^f^Reference category = student.

The difference between the absolute values of these effects is also presented.

As shown in Table 3, most predictors had significant effects on both PMH and NMH, although the effects tended to be stronger for NMH. Gender (female), non-European origin, socio-economic factors (“poor” housing, sickness benefit/disability pension, economic strain), life-style factors (alcohol consumption and risky alcohol consumption, risky gambling, smoking), victimization (humiliation, threat, violence) and suicidal expression (suicide ideation, suicide attempt) had positive crude associations with NMH that were stronger than their negative crude associations with PMH. Factors related to assets (i.e., being employed vs. being a student), interpersonal trust, and community trust had negative crude associations with NMH that were stronger than their positive crude associations with PMH. Participation in societal events had a positive effect on PMH that was larger than the corresponding negative effect on NMH. Healthy eating also had a positive effect on PMH but did not impact NMH.

In the adjusted model, where all effects were controlled for with respect to one other, many presumptive predictors were still significantly correlated with PMH and NMH. However, the predominance of strong associations between the presumptive predictors and NMH was largely extinguished. This was obvious for the factors related to interpersonal trust, threat, violence, and suicide attempts, where the differences between PMH and NMH nearly disappeared. The remaining potential predictors which elevated NMH more than they decreased PMH were female sex, economic strain, risky gambling, humiliation, and suicidal ideation, with the strongest effects associated with suicide ideation (absolute difference − .371) and humiliation (absolute difference − .295). The difference between employed and student status retained the same tendency observed for the crude effects (employment decreased NMH more strongly than it elevated PMH), while the adjusted model revealed that being too ill or disabled for employment reduced PMH more than it elevated NMH, although both effects were significant. Increased age deteriorated PMH in both models and the absolute difference remained, although increasing age had no significant effect on NMH. The only factors that significantly elevated PMH without influencing NMH were participation in societal events and gambling in moderation (but not risky gambling).

## Discussion

In this study, we explored data from a questionnaire with demographic and health related items that had been administered to a study group of more than 41,000 individuals (16–29 years of age, living in Sweden). As a first step, we performed an explorative factor analysis on data from the GHQ-12, which revealed a two-factor model (each factor had six items). The fit of this model was supported by the performance of a CFA, which yielded a significantly better fit than a unidimensional model. The factors were tentatively called “positive mental health” (PMH) and “negative mental health” (NMH). When we investigated associations between GHQ-scores and potential predictors of health, we found a large number of mirror-like associations between PMH and NMH. Specifically, if the potential predictors were positively associated with one of these health concepts, they were also negatively associated with the other. We found that this relationship was the strongest for suicidal ideation and perceived humiliation.

The results of our factor analysis thus seem to support the hypothesis that positive and negative mental health are different constructs. This is consistent with earlier findings [4, 20]. On the other hand, the mirror-like associations between these constructs and the potential predictors fit well with a unidimensional model. It is possible that the results of the factor analysis specifically reflect the wording of the items, rather than influence of the two hypothesized health constructs. Several authors have suggested this interpretation. For instance, Hankins claimed that the bi-and multi-dimensionality attributed to the GHQ [41, 42] is an artefact because of a response bias of the negatively worded items. Additionally, he argued that the GHQ-12 is unidimensional, and that its use is limited to the identification of psychiatric morbidity [43, 44]. Other authors have drawn similar conclusions [45, 46]. For instance, Smith et al. conducted a British population study about ageing, and suggested that item phrasing, item variance, and the level of respondent distress might explain the heterogenous results with respect to the factor structure of GHQ-12.

The associations between possible determinants were stronger for NMH than for PMH. This may simply reflect the fact that the survey was originally constructed to capture determinants of ill-health rather than health. Female sex was related to both of the factors that we examined, and we found that the positive association with NMH was stronger than the negative association with PMH in the fully adjusted model. Most health surveys have found that women report higher NMH-scores and a higher prevalence of diagnoses related to depression and anxiety [47, 48].

We found suicide ideation and humiliation to be the strongest predictors of NMH. Notably, humiliation—reflecting exposure to harassment, bullying, or discrimination, for example—had a stronger association with NMH than exposure to threats or violence. In a Swedish online survey, 24% of young individuals (15–24 years of age) reported several instances of harassment and 15% reported repeated exposure to discrimination based on gender, ethnicity, or sexual orientation, highlighting the magnitude of these problems [49]. Our findings concerning the association between humiliation and mental ill-health outcomes are consistent with findings from many other studies. Indeed, a previous study with a sample of young adults found a dose-dependent relationship between verbal abuse committed by adults and peers and psychiatric symptoms [50]. Additionally, a meta-analysis found an association between peer victimization and indicators of psychosocial maladjustment, such as depression, loneliness, and generalized and social anxiety [51]. Of specific concern is the phenomenon of cyber bullying, i.e. electronic bullying or online social cruelty [52], which especially affects young people. A dramatic growth in the prevalence of cyber bullying has been observed during the last several years [53]. There is also considerable evidence for an association between exposure to bullying and self-harm, violent behavior, and even psychotic symptoms [54]. Thus, efforts aimed at reducing bullying and victimization in childhood and adolescence should be strongly supported, also as a way to prevent psychiatric symptoms [54].

Participation in social events and restrained gambling activities seem to promote PMH without decreasing NMH. Societal participation and playing an active role in one’s social environment are frequently considered to be key determinants of mental health [55–57]. While excessive gambling is commonly associated with poor mental health [58, 59], moderate or strategic gambling may have the opposite effect.

Our data indicate that PMH decreases as age increases in people between 16–29 years old, although we found no age related differences concerning NMH. This phenomenon could be attributed to the prolongation of emerging adulthood with particular hardships in establishing in work, partnership and housing which may diminish PMH but not necessarily influence NMH. In particular, being a student, which is usually seen as a desirable position, was related to higher NMH and lower PMH when compared to being employed. This may be explained by the increase in youth unemployment during the last two decades. As a response to this development, young people may stay in school longer than they would normally be comfortable with.

### Strengths and limitations

One strength of our study is the large population size, which was based on a national sample. Another strength is the focus on the health of adolescents and young adults, as this group experienced a societal shift during the decade preceding data collection [28] that might have contributed to deteriorated health. One limitation is the cross-sectional design of the study, which rules out any possibilities of inferring causation. The high non-response rate should be acknowledged, although declining response rates, especially in cross-sectional studies, have been reported during the past decades. Our attrition rates were similar to those observed in other surveys [60, 61]. To improve the generalizability of the data, upcoming surveys could take various measures to improve response rates, e.g. contacting respondents in advance and personalizing letters and questionnaires [62].

The current study design does not allow any predictions about the strength of the observed outcomes. Thus, further study with a longitudinal approach is required.

## Conclusions

Ultimately, we were unable to determine whether GHQ reflects two mental health dimensions. We cannot rule out the alternative hypothesis that the fit of the two-factor model is related to the wording of items. Based on these findings, we suggest that the GHQ-12 is useful for the purpose for which it was intended, i.e., to detect “psychiatric morbidity” in clinical and population settings. We believe that future investigations of the dimensionality of “mental health/ill-health” needs to use instruments that are specifically adapted for this purpose. The identification of predictors of positive mental health requires more refined measures than those used in our study. Specifically, predictors that are theoretically linked to health rather than to ill-health would be useful. The objective of public mental health is to promote health as well as detect symptoms of ill-health. We suggest that baseline measurements are very important for this goal, and that appropriate instruments, including measures of positive mental health, should be used. The strong association between humiliation and negative mental health is of interest, especially given the general debate regarding the increase of themes of humiliation in western entertainment and media.
